# Direct Versus Indirect Treatment Options of Endodontically Treated Posterior Teeth: A Narrative Review

**DOI:** 10.7759/cureus.67698

**Published:** 2024-08-24

**Authors:** Mai M Alhamdan, Rodina F Aljamaan, Munira M Abuthnain, Shahd A Alsumikhi, Ghada S Alqahtani, Reem A Alkhraiyef

**Affiliations:** 1 Department of Prosthetic Dental Sciences, King Saud University, Riyadh, SAU; 2 Department of Dentistry, King Saud University, Riyadh, SAU

**Keywords:** crown, endo-treatment, final restoration, posterior, tooth structure

## Abstract

One of the controversial topics in dentistry is restoring endodontically treated posterior teeth. Most posterior teeth that undergo endodontic treatment are subjected to a high rate of fracture due to excessive amount of loss of tooth structure. The aim of this review is to evaluate and compare the restorative and prosthetic treatment options to provide clinical recommendations for restoring endodontically treated posterior teeth. Both Medline on PubMed and Google Scholar were utilized for the search. The terms on our keyword list were "crown," "onlay," "endo-crown," "amalgam," and "composite," with the time frame from 1977 to 2024. We also examined the reference lists of potentially relevant papers for any recent review articles. Our analysis examined review articles found through computerized searches, along with relevant citations from the bibliographies of those studies. This review will focus on the dental restorative options and the amount of remaining tooth structure in determining the final restoration of an endodontically treated posterior tooth. This narrative review addresses different treatment options for endodontically treated posterior teeth based on the amount of remaining tooth structure. In addition, it compares the survival rate and the limitations among direct and indirect restorations.

## Introduction and background

Restoration of endodontically treated teeth (ETT) is a challenge that every dentist needs to deal with, and the choice of final restoration is still unclear. Root canal filling, also known as endodontic treatment, is a very common dental operation that involves removing the tooth pulp and replacing it with a root canal filling. It is frequently suggested when there has been irreversible inflammation or necrosis of the pulp, resulting from caries or trauma [1]. When compared to tooth extraction followed by implant placement and root filling, subsequent restoration is the more cost-effective choice [2]. The success of posterior teeth that have undergone endodontic treatment not only depends on the quality of the endodontic procedure but also on the subsequent restorative methods [3,4]. Before choosing the final restorations of endo-treated teeth, we have to consider many factors, such as the amount of remaining tooth structure and the position of the tooth in the dental arch [4]. As in the past, clinically and radiographically acceptable root canal therapy proved successful in avoiding bacterial infiltration and enhancing periapical pathosis healing. Recent studies have provided evidence that the quality of the final coronal restoration is critical to tooth stability [2].

The main purposes of the final restoration of ETT are to avoid bacterial microleakage into the root canal system, restore function, preserve the remaining tooth structure, and maintain the integrity of periodontal health structure and esthetic [5-7]. After root canal therapy is completed, many prosthetic treatment approaches have been recommended. The majority of them involved either direct restorations, such as resin composites and amalgam, or indirect restorations, such as crowns, endo crowns, and endolays. According to different study designs, clinical procedures, assessment criteria, and lengths of postoperative observation periods, conventional root canal treatments have success rates ranging from 40% to 97% [8]. However, failure of the restoration rather than the endodontic procedure itself is the main reason why endodontic treatment has failed [3]. A study concluded that the most important element determining the long-term survival of teeth following RCT is the timely placement of the final coronal restoration [9].

The aim of this review is to evaluate and compare the restorative and prosthetic treatment options to provide clinical recommendations for restoring endodontically treated posterior teeth. This review will focus on the dental restoration properties and the amount of remaining tooth structure in determining the final restoration of endodontically treated posterior tooth.

## Review

Methodology

Both Medline on PubMed and Google Scholar were utilized for the search. The terms on our keyword list were "crown," "onlay," "endo-crown," "amalgam," and "composite," with a time frame from 1977 to 2024. We also examined the reference lists of potentially relevant papers for any recent review articles. Our analysis examined review articles found through computerized searches, along with relevant citations from the bibliographies of those studies.

Amount of the remaining tooth structure

The assessment of the remaining tooth structure is required as a part of the treatment plan for the posterior tooth that undergoes endodontic treatment followed by final restoration [10]. This gives an indication of whether the tooth is restorable or not. A known tooth structure could be damaged by extensive caries, over-preparation of the access cavity, or if the dentist does not follow the proper conservative treatment.

When the tooth undergoes endodontic treatment, it gets exposed to “irreversible chemical-physical changes including dehydration of dentin, reduction of micro-hardness, collagen alteration, effects of irrigants and medicaments, and bio-mechanical changes as the loss of tooth structure and loss of proprioception that will lead to an increase in the chances of dental fracture” [11]. In addition, the remaining tooth structure plays a vital role not only in choosing treatment options but also in long-term survival [12,13,14,15]. There is a direct relationship between the remaining tooth structure and fracture resistance. Greater remaining tooth structure means greater longevity for the teeth [16,17,18].

One author demonstrated that ETT with a remaining coronal structure showed a transfer of tensile stresses to the cervical third of the root's outer surface, lowering the risk of catastrophic failure. Meanwhile, teeth with no coronal structure showed a different distribution of stresses, increasing the risk of irreversible fracture [19].

Kaplan-Meir method has been developed as a criterion-based evaluation, classifying the remaining tooth structure into three types: type I cavity preparation when the remaining wall thickness is at least 2, type II for moderate remaining tooth structure representing class II cavity preparation with no less than two walls with at least 2 mm thickness, and type III for minimum remaining tooth structure as the remaining coronal tooth structure had less than two walls with at least 2 mm thickness. The survival rate of the three types in the first, second, and fifth years were 96%, 88%, and 36%, respectively; the median survival time (95% confidence range) was 3.7 years (2.9 to 4.5 years ) [20]. Another study has been concluded, which found that the lower the remaining tooth structures, the lower the success rate of restorative treatment of ETT [5,21].

Throughout each stage of the treatment, a minimally invasive technique should be used. It is crucial to minimize the removal of healthy dental tissues during endodontic therapy (access cavity preparation and root canal instrumentation) and restoration procedures (post-space preparation, final cavity preparation, and restoration type selection). Currently, it appears that the most successful method for restoring teeth that have had endodontic treatment is by preserving intact coronal and radicular tooth structure and maintaining cervical tissue to create a ferrule effect, which are crucial to optimize the biomechanical behavior of the restored tooth [3]. Moreover, the use of adhesive procedures in the restoration of ETT, which will not undergo full coronal coverage, aids in strengthening the remaining tooth structure and optimizing the stability and retention of the restoration [20].

Ferrule effect

A ferrule effect with 1.5 to 2 mm contributes to the fracture resistance of ETT. A ferrule effect is defined as "a metal collar of the crown surrounding the parallel walls of the dentine extending coronal to the shoulder of the preparation.” The result is an elevation in the resistance form of the crown from the extension of the dentinal tooth structure [11].

A study done by Bacchi et al. in 2019 aimed to examine the influence of the type of the post and ferrule effect on the fracture strength and stress distribution of stress in premolars; it concluded that the influence of the post varies depending on the presence or absence of a ferrule effect [22]. During the assessment of the remaining tooth structure, it is important to consider having a ferrule effect so that we can choose the optimum treatment options with a high success rate and longevity.

Direct restorations as the final restoration 

In dentistry, many types of direct restorations have been used as the final restoration with the endodontically treated posterior teeth, such as resin composites, amalgam, GIC (glass-ionomer cement), and IRM (intermediate restorative material). Many factors should be evaluated before the application of direct restoration because ETT have reduced coronal and radicular tissues because of dental caries, access cavities, intra-radicular procedures, and the presence of a previous restoration [23,24,25].

In Belli et al.'s study (2015), it was suggested that occlusal coverage of ETT with direct restorations is considered imperative for the survival of the tooth [23]. However, the success of the direct restoration of ETT mainly depends on the remaining tooth structure.

In a retrospective study, after endodontic treatment, molar teeth that had maximum tooth structure remaining had a survival rate of 78% at five years; however, severely compromised endodontically treated molars restored with direct composite resin had a survival rate of 18% at five years [20]. A previous study found that over 70% of endo-treated posterior teeth have already lost more than two-thirds of their coronal tooth structure, making a bonded restoration unfeasible [26,27].

After evaluating different types of restorations, they found that amalgam was inappropriate for the final restoration due to the high chance of fracture in ETT as it does not adhere to the tooth structure [23,28].

When using GIC, it was found that the material was brittle and ill-suited as permanent filling material in the posterior region, so it may not be appropriate as a final restoration [23,29,30]. 

On the contrary, resin composite restoration was significantly stronger than the glass ionomer core when measuring the fracture strength [31]. Finally, the study concluded that using composite resin was a good treatment option. However, direct restorations are technique-sensitive with a major limitation of polymerization shrinkage, microleakage, and secondary caries. Recent suggestions indicate that adhesive resin composite restorations may be a viable option for restoring posterior ETT with minimal to moderate loss of tooth structure [32,33].

Post-retained restorations

A majority of ETT exhibit various amounts of remaining tooth structure; in order to obtain a satisfactory retention of the final restoration (direct or indirect restoration), a post may be required [15,23].

A randomized clinical trial (RCT) done by Mannocci et al. in 2002 concluded that “the clinical success rates of endodontically treated premolars restored with fiber posts and direct composite restorations after three years of service were equivalent to a similar treatment of full coverage with metal-ceramic crowns” [34]. Many studies have concluded that using the post is necessary to retain the core, for coronal restorations, and to reduce the risk of root fracture [35,36,37].

Direct vs. indirect restorations

Indirect restorations are now more widely available due to the development of materials with the strength to sustain functional force and aesthetic appearance [38,39,40].

ETT have a better long-term prognosis when the cuspids are covered [11,41]. Based on the findings of this in vitro study, it seems that any type of indirect restoration with cuspal coverage is effective for restoring ETT. The study found that all indirect restorations, such as endocrowns, partial crowns, and full crowns, performed well after being subjected to in vitro fatigue loading [42].

A meta-analysis study conducted by Shu et al. in 2017 compared the success rates of direct and indirect restorations for ETT. The study concluded that indirect restorations, mainly crowns, exhibited higher survival rates in the short term (five years) and medium term (10 years) compared to direct restorations like composites or amalgam. However, there was no significant difference in the short-term (less than five years) restorative success or endodontic success between the two types of restorations. The study emphasized the need for high-quality clinical trials, particularly well-designed RCTs [3].

An endocrown is considered an alternative option to conventional post, core, and crowns as it preserves root tissues and limits internal preparation of the pulp chamber due to its anatomic shape; it also has good aesthetic results and lower chair-side time [43,44,45]. Endocrowns are considered less invasive and more conservative options for the treatment of ETT although full-coverage crowns are still more common than partial-coverage crowns [46]. They are also considered more cost-effective with a less willingness-to-pay (WTP) value compared to a complete crown, which has a higher WTP threshold, but it became a more cost-effective restoration [47]. 

According to a systematic review by Lenz and Bacchi (2024), the performance of endocrowns was similar to or even greater than that of a traditional post, core, and crown [48]. However, endocrowns should be restricted only to molars [49,50].

Using endocrowns as a final restoration in posterior ETT is considered when a sufficient amount of tooth structure is presented to support the adhesive restoration. Studies have found that using endocrowns over post-core-supported crowns shows better marginal adaptation and less marginal leakage and is considered a reliable alternative to post-core-supported crowns [50,51]. However, there are insufficient studies supporting the preference for endocrowns over conventional ways of restoring posterior ETT in terms of survival rate and performance [5,45].

As is known, direct composite resin restorations are less expensive and can be created in just one visit, requiring little to no tooth tissue removal. A more rigid material that can be fabricated outside of the oral cavity, giving more control over anatomy, can be used to create an indirect restoration [49].

Research has demonstrated that premolar ETTs with one or two missing marginal ridges achieve similar clinical success rates when restored with either crowns or resin composites. However, the clinical significance of this approach is still under debate and requires additional investigation [52].

Immediate replacement of restoration is a critical thing that we should keep in mind [53,54]. Cusp fractures in treated teeth, regardless if they undergo root canal therapy or not, are common findings in dentistry. A high impact of the forces comes from biting on a hard item, which is considered the most common cause, but tooth structure, restoration type, and size all play major roles [55].

In 2016, Isaac Pratt conducted a retrospective study to examine the impact of different coronal restorative treatments on the survival rate of ETT. The study found that teeth restored with composite/amalgam buildups were more than twice as likely to be extracted compared to those restored with crowns. In addition, the timing of crown placement after root canal treatment significantly affected the survival rate, with teeth receiving crowns four months post-treatment being almost three times more likely to be extracted than those receiving crowns within four months of treatment [56].

A review conducted by Fedorowicz et al. in 2012 concluded that there is inadequate evidence to support or dispute the effectiveness of conventional fillings over crowns in restoring root-filled teeth [57]. A systematic review done in 2016 by da Veiga et al. concluded that neither direct nor indirect restoration has superiority in the longevity of the restoration [35].

Indirect restorations

According to a systematic review done by Stavropoulou and Koidis in 2007 on single crowns on ETT, teeth covered with crowns had a superior survival rate of 81% after 10 years compared to RCT teeth without crown coverage, which was 63% after 10 years. However, it should be noted that the survival rate for teeth without crown coverage is quite pleasing in the first three years, which was 84%, while there is a noticeable decrease in survival after this time [8].

Moreover, another systematic review conducted in 2017 on the survival rates against fracture of endodontically treated posterior teeth restored with full-coverage crowns or resin composite restorations found that survival rates against fracture of posterior ETT restored with either full-coverage crowns or direct resin composite restorations were not significantly different in the teeth with minimal to moderate loss of tooth structure [54].

A study done by Polesel in 2014 regarding the restoration of endodontically treated posterior tooth concluded that direct adhesive restorations, indirect bonded restorations, and traditional full crowns are the main treatment options for a single endo-treated posterior tooth, but with advanced developments in new adhesive techniques, it starts to help in terminating the use of full crowns, which help partial procedures in providing conservative treatment with the same amount of protection [11].

A systemic review conducted in 2021 concluded that for short-term restorative success, low-quality evidence suggested no difference between the direct and indirect restorations. However, the study suggests doing clinical trials that control for the amount of coronal tooth tissue and other baseline characteristics and assessing the influence of the type of restoration on the survival and restorative success of endodontically treated posterior teeth [58].

Partial coverage all-ceramic crowns can offer a conservative substitute for full coverage restorations on posterior teeth. An RCT revealed that in three years, the survival rate of restoration and tooth was 93.3% for premolars and up to 100% for molars with 50% or more coronal residual tooth structure following restoration with partial coverage lithium-disilicate crowns [59]. In a retrospective study, lithium disilicate glass-ceramic partial-coverage crowns were used to restore 121 morphologically compromised posterior endodontically treated teeth. The estimated survival rate of the teeth was 99% for seven years, whereas the estimated survival rate of the restorations was 94.8% for five years and 92.8% for seven years [60]. Therefore, functional needs and the amount of residual tooth structure should be taken into consideration when deciding whether to use a crown or a partial-coverage restoration [3]. A retrospective cohort study was conducted by Steven and Daniel using a database of 280 patients, which evaluated the effect of crown placement on root canal-treated tooth survival. It found that endodontically treated teeth not crowned after obturation were lost at a six times greater rate than teeth crowned after obturation [61].

Recommendations and future directions

Although this review has covered many studies conducted to evaluate the behavior and survival rates of different restorative options used to restore endodontically treated molars, the authors still recommend commencing more targeted longitudinal in vivo studies to evaluate the success rate of endocrowns on endondontically treated molars and comparing the performance of endocrowns versus conventional crowns. The findings should support the best final decision to restore endodontically posterior teeth.

Limitations

There are limitations in finding long-term studies that evaluate and compare the success and survival rates of direct restorations, indirect restorations, conventional crowns, and endocrowns in endodontically treated posterior teeth. Moreover, narrative reviews have their own limitations of subjectivity potential bias and challenges in data interpretation.

## Conclusions

One of the controversial topics in dentistry is the restoration of endodontically treated posterior teeth. Posterior teeth that undergo endodontic treatment are subjected to a high rate of fracture due to an excessive amount of tooth structure loss; therefore, restoring them with an appropriate final restoration is crucial. Direct restorations could be considered when there is sufficient tooth structure; however, they are technique-sensitive and have a major limitation of polymerization shrinkage, microleakage, and secondary caries. The application of indirect restorations with full cuspal coverage has reported high survival rates in short-term studies; however, the need for a ferrule effect and the time of placement of the restoration after RCT must be considered for the survival of the restoration. Endocrowns have been identified as an alternative option to conventional crowns in molars under certain conditions. However, there are limitations of long-term studies in determining the success rate between crowns and endocrowns in molars.

In conclusion, the superiority of the survival between direct and indirect restorations has not yet been concluded with high-quality studies. Until more promising and high-quality studies provide evidence-based decisions, dentists will continue to base their decisions, case by case, on clinical experience, the amount of remaining tooth structure, the presence of ferrule, and the properties of each restoration.
